# Maternal Harsh Physical Parenting and Behavioral Problems in Children in Religious Families in Yemen

**DOI:** 10.3390/ijerph16091485

**Published:** 2019-04-26

**Authors:** Khadija Alsarhi, Rahma, Mariëlle J. L. Prevoo, Lenneke R. A. Alink, Judi Mesman

**Affiliations:** 1 Education and Child Studies, Leiden University, 2333AK Leiden, The Netherlands; r.rahma@fsw.leidenuniv.nl (R.); prevoomjl@fsw.leidenuniv.nl (M.J.L.P.); ALINKLRA@FSW.leidenuniv.nl (L.R.A.A.); MESMANJ@FSW.leidenuniv.nl (J.M.); 2 Faculty of Public Health, Hasanuddin University, Makassar 90245, Indonesia

**Keywords:** harsh physical parenting, religiosity, Yemen, child behavioral problems, discipline, slums, video observation

## Abstract

The present study examined maternal religiosity as an underlying cultural factor in the effect of harsh physical parenting on child behavioral problems. Data was collected via a discipline observational task, religiosity-based vignettes, and a questionnaire in a group of 62 mothers and their children in slum areas in Yemen. Moderation and mediation models were tested, where the role of maternal religiosity as a predictor and a moderator in the association between harsh physical parenting and child behavioral problems was explored. Findings showed no direct association between harsh physical parenting, maternal religiosity, and child behavioral problems. However, maternal religiosity was found to significantly moderate the relationship between harsh physical parenting and child behavioral problems such that the positive association between harsh physical parenting and child behavior problems was stronger when parents were more religious. Implications of the moderating role of maternal religiosity on the association between harsh physical parenting and child behavioral problems are discussed.

## 1. Introduction

Harsh physical parenting, such as hitting and spanking, is associated with range of adverse negative child outcomes, such as behavioral problems, lowered self-esteem, and adverse mental health [1,2,3]. However, to date most studies have mainly examined the behavioral and emotional outcomes of harsh physical discipline in white, two-parent, middle-class families [4], with more recent attention being given to African American families [5,6,7,8]. Because the cultural normativity of harsh parenting can moderate the association between this type of parenting and child outcomes [9], expanding this area of study to families from different backgrounds [10,11] with attention to relevant cultural factors such as religiosity is needed. Maternal religiosity in relation to harsh parenting and child outcomes has revealed mixed results in the literature. While a prominent part of the literature showed that maternal religiosity is related to harsher parenting [12,13] and more negative child outcomes such as antisocial behavior, negative psychosocial outcomes, child problem behaviors, and academic failure [10,14,15,16], other studies have shown positive effects of maternal religiosity on parenting and child outcomes, for example, lower rates of less child abuse [17,18]. However, the presence of both positive and negative effects of maternal religiosity on parenting underlines the importance of religiosity in trying to understand harsh parenting and child development, especially in cultural contexts where religion is a salient part of life. However, to date, most studies have examined maternal religiosity among English-speaking populations in Western societies (e.g., the United States), focusing on Judeo-Christian religions [18,19]. The current study explores the role of maternal religiosity as a predictor and a moderator in the association between harsh physical parenting and child behavioral problems in low-income Muslim families in Yemen.

### 1.1. Harsh Physical Parenting and Child Behavioral Problems

Parental discipline tactics have received great interest from researchers, with a particular focus on the implications of harsh physical discipline on child development [20]. Harsh physical discipline is regarded as the parents’ attempt to control or punish a child using physical forms of punishment, e.g., pinching, hitting, spanking etc. [3]. Harsh physical discipline has been commonly associated with a negative trajectory in children’s development [16,21], including child internalizing and externalizing problems [22,23,24,25,26,27].

Even though parents generally use physical punishment with the intention to end inappropriate or undesirable behavior and to encourage acceptable behavior from their children, physical punishment is instead known to have negative child outcomes [14], for example increasing instead of decreasing the undesirable behaviors [28]. Earlier studies found that the more physical punishment is used by parents, the more aggressive their children become in the long-term [5,16]. Another study showed that maternal harsh parenting distinctively contributes to negative behavioral adjustment in children [29].

However, research also showed that the effects of maternal physical discipline on child outcomes may not be universal, as contextual factors such as culture, ethnicity, and religion can play a role in that relationship [30]. For example, cultural normativity was found to play a moderating role in the relation between physical discipline and child adjustment [9]. In other words, in cultures where physical discipline was more normative, it was found that more frequent use of physical discipline was less strongly associated with adverse child outcomes [9]. In another study, ethnic group differences played a moderating role as well in the relation between physical discipline and child adjustment [5]. In this study, it was found that physical discipline was less strongly associated with child behavior problems for African-American children in comparison to European-American children. In addition, religion, which is possibly one prevalent aspect of culture [6], was also found to have a role in harsh parenting and child outcomes, but has received limited attention from social scientists [31]. It is the aim of this study to investigate the role of religion in harsh parenting and child behavioral outcomes.

### 1.2. Religion and Maternal Religiosity

Although religion can have a significant impact on parents’ attitudes toward physical discipline [32] and thus on parenting behaviors, research on how religion may shape parent–child relationships has received only limited consideration by social scientists [18,31]. That said, it is even more unfortunate that most of the available research on religion in relation to parenting has mainly involved English-speaking populations in Western societies (e.g., the United States), focusing on Judeo-Christian religions [18,19]. Results from the available literature on parenting and child outcomes in religious versus non-religious families have shown mixed results, where some studies presented positive effects of religion while others presented negative effects. For example, research reveals that religious parents use corporal punishment [33] and engage in harsher discipline more often than non-religious parents [34]. On the other hand, research also shows that children of religious parents show more empathy and sensitivity in everyday life [6] and more positive psychosocial adjustment [35] than children of non-religious parents. However, these findings on religious and non-religious families reveal only part of the picture on religion and parenting. In the available literature, we have found fewer studies that shed the light on differences in parenting in samples of religious families who differ in their level of religiosity (18; 28; 33; 7; 36; 17).

While religion refers to an organized socio-cultural-historical system of beliefs that connects people to an order of existence, religiosity is a measure of a person’s devotion and involvement with a religion [10]. Part of the literature has highlighted that a higher level of religiosity is related to harsh parenting and negative child outcomes, while other studies have revealed the opposite. For example, one study showed that physical punishment was used by parents with conservative scriptural beliefs more recurrently than by parents with less conservative theological views [12]. In their meta-analysis of studies from 1980 to 1999, Mahoney et al. [18] showed consistent evidence that conservative Protestant parents do spank young children more often than non-conservative Protestant parents. In another community sample, it was shown that biblically liberal mothers spanked their children less than biblically conservative mothers [34]. Other studies explicitly stated that in general, conservative Protestants exhibit more cohesive support for harsh parenting than parents who are less conservative [7,32]. On the other end of the spectrum, research also presented some positive effects of religiosity on parenting. For example, across a 17-year period, one study showed that in religious families in which parents rarely attended church services, young children were more than twice as likely to be abused physically as were children whose parents attended church regularly [36,37]. A meta-analysis revealed that maternal religiosity was related to positive parenting and child outcomes, even though parents used harsh parenting techniques like spanking [18]. The positive child outcomes might be explained by the consistency, confidence, and provision of clear limits by parents with strong religious beliefs [18]. Also, mothers with high religious involvement displayed less child abuse potential [17].

Moreover, previous literature revealed mediational models where religiosity played an important role in physical punishment and child behavioral problems. Mediational models showed that conservative Protestant parents who exhibited more religiosity showed excessive harsh parenting which, in turn, increased the probability of children’s mental health problems; e.g., rebelliousness, fearfulness and guilt [12], and child problem behaviors [10]. Another unique mediational study which included four different religions (Catholicism, Protestantism, Buddhism, and Islam) showed that maternal religiousness is associated with harsher parenting which, in turn, increased child problem behaviors [10].

Interestingly, it was also found that religiosity played a moderating role in the relationship between harsh parenting and child behavioral outcomes. One study showed that children whose mothers were more religious exhibited minimal adverse effects of physical punishment in comparison to their counterparts whose mothers were less religious [38]. This could be due to the normative support among religious communities for harsh discipline and the fact that some family members and peers have been exposed to harsh parenting. In comparison to their counterparts in other settings where harsh discipline may be condemned, it is less likely that children of religious families perceive this practice as denounceable. In fact, they might consider it as appropriate, and an indicator of maternal involvement and concern [38].

In summary, our literature review shows that maternal religiosity is related to both parenting and child outcomes, and may play a significant role in family life. However, it is still unclear whether those findings from previous research can be generalized to low-income families or families from non-Western cultural backgrounds [39]. Therefore, it is the intention of this study to add to the above literature on religiosity and low income families by shedding light on the role of religiosity in the association between harsh parenting and child problems in a low-income Muslim sample of families who live in slums in the north of Yemen.

### 1.3. Maternal Religiosity in Yemen

Islam is the main religion of Yemen, and the constitution of this country states that Islamic law is the source of all legislation [40]. The percentage of Muslims in Yemen is 99% [41]. The family unit is a crucial component of Islam, in which parents and children are given due significance. For example, the holy book, the Quran, highlights the position of children by describing them as an adornment of the worldly life [42]. The prophet Mohammed emphasized the parents’ important responsibility towards their children when he said

“Every one of you is a shepherd and is responsible for his flock.”

[43]

On the other hand, the holy book highlights children’s respect and obedience to their parents by asking children to:

“Speak to them (parents) a generous word. And, out of kindness, lower to them the wing of humility, and say: ‘My Lord! Bestow on them Thy Mercy even as they cherished me in childhood’”

[44]

Based on the teachings above, parents feel the responsibility bore upon them by religion to raise their children in the right way and at the same time raise their children so they eventually become obedient. When faced with parenting challenges, parents sometimes tend to use their authority that is driven from religious text to exercise their harsh parenting techniques. For example, to teach their children to pray, parents are authorized to hit their children as shown by the following quote from the Hadith: “The Messenger of Allah said: ‘Command your children to pray when they become seven years old, and beat them for it [prayer] when they become ten years old’” [45] (p. 366). However, it is essential here to mention that there is controversy in the Muslim countries on the accuracy of sayings attributed to prophet Muhammed. In order to know whether the Hadith is strongly or weakly attributed to the prophet Muhammed, Hadith scholars have created a classification of all Hadiths to determine how authentic (or not) the Hadiths are [46]. However, strong and weak Hadiths have become integrated in the Muslim culture, where people refer to and apply those Hadiths to their different aspects of life [47]. The above mentioned Hadith on hitting children to pray is one of the more controversial Hadiths.

Interestingly, in other monotheistic religions such as Christianity and Judaism, the parent–child relationship is built around the same principles. For example, in Christianity, parents are reminded of the valuable position of children; they are considered a heritage from the Lord, the fruit of the womb, a reward [48]. Children are asked to obey their parents in everything to please the Lord [49], and the parents of a stubborn and rebellious son who does not obey them should have him stoned to death [50]. Similarly, in Judaism, parents are asked to support their children, particularly daughters [51]. In commandment number five, children are asked to honor their parents [52], and in terms of discipline, parents are asked not to spare the rod [53].

Harsh parenting in Muslim Arab families can also be driven from cultural beliefs that the father is the owner of the child and it is his right to raise the child in the best way he considers, whether it is violent or not [54]. Another cultural proverb in the Arabic culture that encourages harsh physical punishment is: “punishment awaits those who disobey” [54]. In summary, it can be said that in the Muslim Arabic context, harsh parenting is embedded in culture, where religion is considered an integral aspect.

In Yemen, the culture of harsh parenting is prevalent, as more than 80% of children are reportedly subjected to physical punishment by adults including parents [55]. Al-Dabhani [56] paralleled this in his research finding on harsh punishment, where 82% of the parents in his study believed that using physical punishment was correct, and used punishment upon misbehavior of their children. Alyahri and Goodman [57] also found that Yemeni children with behavioral and emotional disorders were 2–3 times more likely to experience harsh parenting [57]. With that taken into account, the Yemeni context is a particularly interesting country in which to scientifically test the role of maternal religiosity in harsh parenting and child behavioral problems.

To extend the literature on harsh parenting, child development and religiosity, it is the purpose of this study to explore the relationships between harsh physical parenting, child behavioral problems, and maternal religiosity in low-income Muslim families who live in slum areas in Yemen.

### 1.4. The Current Study

The current study is guided by the following hypotheses. Firstly, maternal harsh physical parenting, maternal religiosity, and child behavioral problems are interrelated. Secondly, maternal religiosity plays a role in maternal harsh physical parenting and child behavioral problems in two potential ways: (1) Maternal religiosity is a predictor of harsh physical parenting, which in turn leads to child behavioral problems; and (2) Higher maternal religiosity moderates the effect of maternal harsh physical parenting on child behavioral problems.

## 2. Materials and Methods

### 2.1. Recruitment

A local Yemeni non-governmental organization (NGO), whose aim was to improve the conditions of the poor people through the achievement of social justice, carried out the recruitment and data collection. The main reason for selecting this NGO was that it had been already working in the selected slum settlements in the Taiz governorate. The agreement stipulated that the NGO would terminate data collection for this study at the same time that they would terminate their own work for security reasons because of the war taking place in Yemen. Indeed, during the data collection the NGO decided to terminate their work for security reasons, and therefore the data collection was terminated as well. The head of the NGO was trained online by the first author for recruitment and data collection. After the training, four pilot family field visits were conducted by the local NGO to pilot the instruments and procedure.

### 2.2. Instruments

After the completion of the pilot visits, a home visit procedure was initiated. The study instruments included a questionnaire, a discipline-task video observation, and a vignette experiment. The observational task was adapted to fit the cultural context of the participants based on the NGO’s feedback, which was mainly on the type of toys that were piloted. Toys that did not fit within the slum context in terms of their sophistication were changed to simpler toys that were still attractive to the children to play with. The questionnaire was also adapted language-wise as some words had to be changed to the very local dialect of that part of the city. Also, the scale of the measures had to be cut down from 5-point scale to 3-point scale to elicit more accurate answers from the participants. Finally, for the observation method, tools had to be adapted to fit the local context more precisely.

### 2.3. Procedure

For the launch of the data collection, the head of the NGO, along with her assistant, first visited families to brief them on the project and to ask mothers for their informed consent to participate in the research. Families who had agreed to participate were then visited again for data collection. To recruit more families who did not belong to the NGO’s existing slum network, the NGO worked with community facilitators—women from the local community who played a facilitating role between the NGO staff and the mothers to be recruited.

Data collection was carried out at the participants’ homes as mothers in Yemen spend most of their daily life at home and are more at ease indoors than outdoors. Informed consent forms for participation in the study and for the permission of audio and video recording of parts of the home visit were signed by the literate participants. Consent forms were read out loud for those participants who could not read or write and they were videotaped while giving their consent. Compensation for the home visit was given by means of a small gift at the value of US$6.50 to all participating families. However, in the very deprived families, the NGO found it more appropriate to buy food items worth €5.50 as a gift. The study protocol was approved by the Ethics Committee of the Institute of Education and Child Studies, Leiden University.

The home visit took about two hours, including the structured discipline-task observation, vignette experiment, and questionnaire. The questionnaire that was carried out in an audio-taped interview format due to the illiteracy rate among women in Yemen, which is 65% as compared to 27% among men [58]. The questionnaire included questions on the families’ background, demographic information, socioeconomic conditions, religion, and child behavioral problems. Questions were translated into Taizi, the local dialect of the Arabic language, and then back-translated to English by a local translator to correct any translation errors across cultures.

### 2.4. Participants

Sixty-two low-income mothers and their 2–6-year-old children participated in this study. The sample consisted of families who lived in an urban slum (71%) or in a rural slum area (29%) in Taiz governorate in the southwest of the Republic of Yemen. Inclusion criteria for mothers and their children were as follows: (1) families had to have been living in the selected urban or rural slum areas for at least six months at the time of data collection; (2) mothers had to have at least one child between 2 and 6 years of age of which they were the biological mother; and (3) families had to be Muslim. Mothers or children with significant physical and/or mental health problems were not included as per the exclusion criterion.

Most of the mothers did not know their age, so obtaining a mean maternal age was difficult. Out of 62 mothers, only three knew their birth month and year, while the rest either said they did not know or provided imprecise or unreliable answers, such as “I say 28 or 29. Actually I can say 27. I don’t have a birth certificate” or “I don’t know. Well, you can write 40”. More than 50% of participants had no education at all (53% illiterate). For the remainder the educational level varied between primary school and secondary school level, while only 5% had a college degree. Most of the mothers in the sample were married (89%), 5% were divorced, and 7% were widows. The mean child age was 38.69 months (SD = 10.09). Fifty-two percent of the participating children were girls. The number of children in the participating families varied: 16% of the target children were the only child in the family, 21% had one sibling, 21% two siblings, 16% three siblings, and 26% of the target children had four or more (up to ten) siblings. None of the mothers had a job, while out of the 55 two-parent families, 36 fathers (58%) had jobs. Almost half of the sample (42%) had no monthly income, 18% had a monthly income below 25,000 Yemeni Rials (YER1000 = US$3.54 at the time of the data collection), 21% above YER25,000, and 19% did not know their monthly income or did not want to share that information with the data collectors.

### 2.5. Measures

*Maternal harsh physical interference.* Maternal harsh physical interference was measured in a 5-min disciplinary “prohibition” context [59]. Parents were asked to put a set of culturally appropriate attractive toys in front of their children on the floor. The parents’ task was to ensure that their children did not play with or touch the toys. After 2 min, children were allowed to play for another 3 min with only the least attractive teddy bear. Maternal harsh physical interference was coded and referred to the mother’s harsh attempts to make the child clean up or stop the child from touching the prohibited object. It also referred to any harsh maternal physical interfering behavior that was meant to reinforce a prohibition/commandment. Harshness is shown by using unnecessary force, which causes a bigger physical impact on the child, e.g., hitting or slapping the child, harshly pulling the child’s arm away from the toys, harshly grabbing toys out of the hands of the child, pinching the child’s arm, or shaking the child. The scale ranges from 1 (no harsh interference) to 5 (predominant harsh interference). This variable was dichotomized because of limited variability. In the dichotomized scale, we recoded the scores so that a mother who was not harsh (score 1) was scored as 0 and a mother who was scored as harsh (2–5) was scored as 1. Video observations were independently coded by two trained coders. The intercoder reliability (intraclass correlation coefficients, ICC) for the two coders was 0.80.

*Maternal Religiosity.* We measured maternal religiosity using three items: two vignettes (one involving a boy, one involving a girl) explicitly about religious transgressions, and one open-ended interview question about the goal of parenting in general, without explicitly referring to religion. These three items (all scored 0 or 1) were combined by means of a sum score, with a possible range of 0 to 3.

*Religiosity Vignettes.* Rossi’s factorial survey method [60] is a technique that uses vignettes—brief pieces of text to elicit individuals’ beliefs and judgments. Vignettes are usually short stories of people and their behaviors that participants are asked to respond to and report how they would feel or what they would do themselves in that given situation [61]. Vignettes are a useful method to elicit opinions on sensitive topics for which participants might avoid or not feel comfortable to discuss their own situation or opinions [62]. In this study, two vignettes involving a boy and two vignettes involving a girl were constructed to elicit comments on maternal religiosity, e.g., “Your relative/neighbor tells you that her son who is 10 years old refuses to go with his dad to Friday’s prayer in the mosque. What would you advise her to do?”. Each participant was randomly assigned one of the two vignettes involving a boy and one of the two vignettes involving a girl. Participants’ answers were audiotaped, transcribed, and translated. Maternal religiosity in each vignette was coded as 0 = religion not mentioned and 1 = religion mentioned in the context of a solution for the transgression described in the vignette: “praying”, “prayers”, “God”, “God’s will”, “the prophet”, “what the prophet said”, “Islam”, “Muslim”, “religious sect” (e.g., Salfi), “afterlife”, “Quran”, “parent obedience”, “judgement day”, “heaven”, “hell”, “God’s guidance”, “sin”, “punishment”, “prohibited”, “bad deeds”, “Satan”, “religion”, and “reward”. Scores on the two vignettes were then combined (sum score) for the religiosity vignettes. Answers in which mothers’ mention of religion was only a repetition of the words used in the vignette were not coded as mentions of religion. Intercoder reliability was 0.79.

*Religiosity-Based Parenting Goal.* Parenting goals in general refer to what parents hope to accomplish through their interaction with their children and are believed to guide parenting behavior [63]. Participants were asked about the goal of their parenting in an open-ended question “In your opinion, what is the goal of parenting?”. Responses were audiotaped, transcribed, and translated. A maternal religion-based-goal score of 1 was assigned if a mother’s answer was based on religious beliefs or influenced by religious beliefs such as “God”, “Quran sayings”, “God will reward me”, “religion”, “good deed”, “pray”, “praying”, “prayers”, and “fasting”. If religion was not mentioned, this was scored as 0. Intercoder reliability was >0.99.

*Child Behavioral Problems.* To measure children’s behaviors, emotions, and relation-ships, all mothers were administered the Strengths and Difficulties Questionnaire (SDQ). The SDQ was translated by the first author into Arabic, adopted by the NGO head into the local dialect of Taiz city and then was back-translated to English by a local translator to avoid translation errors across cultures. The SDQ consists of 25 items, with five subscales: emotional problems, peer problems, behavioral problems, hyperactivity, and prosocial behavior [64,65]. Each subscale includes five questions with 3-point response scales (“Not true” = 1, “Somewhat true” = 2, and “Certainly true” = 3). Ten of the 25 items are positively worded “strengths”, and were reverse scored if they contributed to the emotional, behavioral, peer, prosocial, or hyperactivity subscales. The overall reliability of the SDQ turned out to be low with a Cronbach’s alpha of 0.47. Therefore, items that were suppressing the alpha most were deleted one by one until an acceptable alpha level of 0.60 was reached [66]. Based on this approach, items 4, 13, 14, 17, 21, and 25 were deleted. From the mothers’ answers to these items it was apparent that questions were too difficult for them to understand or did not fit their cultural context in terms of what they refer to, e.g., “Can stop and think things out before acting” or “Sees tasks through to the end, good attention span”. The average score on the 19 items was used in the analyses.

### 2.6. Statistical Analyses

Pearson’s correlation analyses were conducted to test the interrelations among all variables. If criteria were met, mediation analyses would be performed to identify the role of maternal religiosity as a predictor of harsh physical parenting which in turn leads to child behavioural problems. According to Baron and Kenny (1986) for a mediation to be established, there are four required criteria: (1) the predictor variable should be significantly correlated with the outcome variable; (2) the predictor variable should be significantly correlated with the mediator variable; (3) the mediator variable should be significantly related to the outcome variable controlled for the predictor; and (4) the association between the predictor and the outcome variable significantly weakens when taking into account the mediator (67). Finally, a moderation analyses was conducted using SPSS process model (IBM, Armonk, NY, USA) to test the moderation role of maternal religiosity in the association between harsh physical parenting and child behavioural problems.

## 3. Results

Descriptives of all variables are shown in Table 1. Maternal harsh physical parenting was observed in 23% of the mothers. The average on maternal religiosity was 1.05, indicating that on average mothers mentioned religion once in their response to the two vignettes or the parenting goal question. The mean score for child behavioral problems was 2.00 which meant that on average children showed moderate levels of behavioral problems. We then explored the interrelations between maternal harsh physical parenting, religiosity, and child behavioral problems by Pearson’s correlations (see Table 2). A positive correlation between the sum score of the boy-based and girl-based vignettes and the parenting goal measure was found; *r*(60) = 0.27, *p* < 0.001. Mothers who mentioned religion in the vignettes also referred to religion in their response to the parenting goals questions. Furthermore, no significant interrelations between maternal harsh physical interference, maternal religiosity or child behavioral problems were found. The lack of significant associations between these variables means that the basic criteria for testing mediation [67] were not met. Therefore, mediation analysis was not carried out.

### Maternal Religiosity as A Moderator

**Figure 1:**
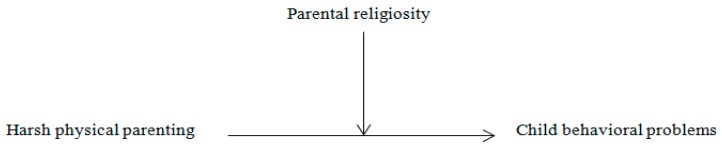
The moderating role of maternal religiosity in the relations between harsh physical parenting and child behavioral problems.

**Figure 2:**
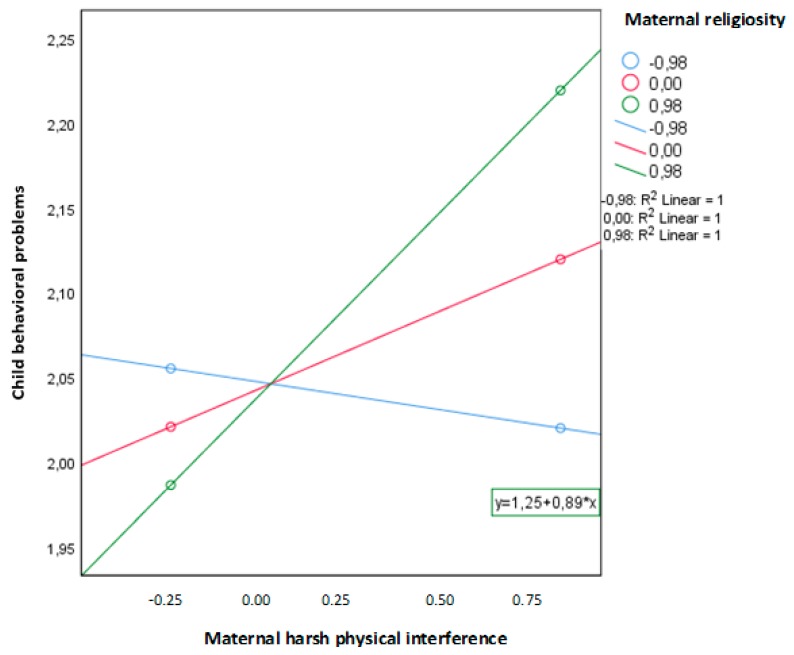
Moderating effect of maternal religiosity for the relation between maternal harsh physical parenting and child behavioral problems.

To test the hypothesis that maternal religiosity moderates the relation between maternal harsh physical parenting and child behavior problems, a hierarchical multiple regression analysis was conducted. In the first step, two variables were included: maternal harsh physical parenting and maternal religiosity. These variables did not account for a significant amount of variance in child behavior problems (*R*^2^ = 0.060, *F*(3,55) = 2.116, *p* = 0.109). However, the interaction term between maternal harsh physical parenting level and maternal religiosity did explain a significant proportion of the variance in child behavior problems (*b* = 0.014, SE = 0.065, *p* < 0.05) (Figure 1). This suggested that the association between maternal harsh physical parenting and child behavior problems depended on the level of the mother’s religiosity. Figure 2 illustrates this interaction effect, showing the change in the expected probability of child behavioral problems by maternal harsh physical parenting for maternal religiosity. Higher levels of maternal harsh physical parenting were related to more behavior problems in children only when mothers had medium or high levels of religiosity.

## 4. Discussion

The current study examined the potential role of maternal religiosity in harsh physical parenting and child behavioral problems in low-income Muslim families in Yemen. Findings showed a moderation effect of maternal religiosity in the association between harsh physical parenting and child outcomes. Children whose mothers showed a higher level of religiosity exhibited stronger adverse effects of harsh punishment in comparison to their counterparts whose mothers showed lower levels of religiosity.

Our results of the stronger adverse effects of harsh physical parenting on child behavioral problems in children whose mothers showed higher level of religiosity were in contrary to previous literature (38). From our study one can speculate that the strength of the association between harsh physical parenting and child behavioral problems thus depends on the religiosity context in which it operates. It might be that more religious parents invoke God to validate their harsh punishments, consistent with the notion “The pleasure of Allah (God) is in the pleasure of the parents, and the displeasure of Allah is in the displeasure of the parents” [68]. When such phrases are used by parents in discipline situations, children might feel shame and fear after harsh discipline as they might consider harsh physical parenting as evidence of parental rejection [69], and therefore God’s rejection. There is evidence that feelings of shame are related to increased risk of internalizing problems such as psychological distress in children [70] and increased anger, which in turn is associated with more behavior problems [71]. In our sample, religious invocations in discipline situations were evident in some mothers’ answers to the religiosity vignette questions. For example, in response to a vignette about what a relative should do with her daughter who refuses to wear the traditional cover up garment when going out, one mother said “She should tell her daughter that Allah will not forgive you or absolve you. In other words she should scare her. If it doesn’t work then she should hit her”. In another example where a daughter refuses to pray, one mother said “She should be strict with her daughter in a way that she can infect her with fear and make her scared so she prays. She should say to her “my daughter, maybe Allah will take you from me or you might get sick or he will put you in big trouble if you don’t listen to me and pray”. If she doesn’t listen then she should hit her”.

The absence of a direct association between maternal religiosity and harsh physical parenting in our sample comes in line with previous literature where it has been shown that the effects of maternal physical discipline on child outcomes may not be universal because of contextual factors such as culture, ethnicity, and religion. Our results might be explained by the general level of religiosity of those who live in slums, which is often not as more apparent as it is among the middle class population [72]. Middle-class families not only have the benefit of being able to read and write, being able to educate themselves on religion either from direct sources like books or schools, but also are active citizens who have the time and financial resources to participate more fully in the political role of religion in their lives and their societies. In contrast, learning about religion and applying it in their day-to-day parenting methods might not be on the top list of concerns for the urban slum poor who are busy struggling to make ends meet for basic survival and concrete concerns [72]. It is also important to mention that in our sample, other contextual factors could have played a role in maternal harsh physical parenting, such as the current war condition that Yemen is going through. Taken together, these findings serve as a reminder that links between similar constructs may be different for families in different contexts [69].

A point of strength of this study is its assessment of religiosity. Because socially desirable answers are more common in collective cultures, religious groups, minority groups, and women [73,74], and these characteristics are all applicable to our sample, our religiosity measures were designed to elicit answers indirectly to avoid social desirability as much as possible. Our religiosity questions were presented in the form of vignettes, which are a useful method to elicit opinions on sensitive topics [62] implicitly and explicitly. In some of the vignettes, religion was mentioned explicitly while in other vignettes it was mentioned implicitly by presenting themes which were related to religion. Moreover, all vignettes presented a situation that was happening to a relative rather to the participant herself. This was done intentionally to decrease the risk of socially desirable answers; participants are expected to feel more comfortable in giving their true opinion about a situation that concerns someone else rather than themselves. The other question of assessing maternal religiosity was through asking an open question on the goal of parenting without mentioning religion. This allowed parents who found religion of importance in their parenting, to refer to it without being prompted.

A limitation of this study lies in the observation of discipline. It should be acknowledged that this study is the first observational study of maternal harsh parenting in Yemen, in a particularly reserved culture where filming women is an issue. This challenge was overcome by the study team [75]. However, for future studies, more attention should be given to the cultural adaptation of the task. Despite efforts to select task toys that should fit within the slum context, we noticed that the toys were clearly both very unfamiliar and very attractive to mothers and children alike. Some mothers could not stop themselves from playing with the toys and therefore got distracted from the task. This also made some children more frustrated that they were not allowed to play with those attractive toys while their mothers could. For future studies, toys that are more common in the local context or even an entirely different paradigm that is more ecologically valid should be explored. A more ecologically valid way could be to ask the parent about an object in or around the house that the child always wants to touch but is not allowed to, and use that as a starting point for the observation of discipline. The last author, Judi Mesman, has tried this in rural Iran (in a yet unpublished study) and this strategy worked well.

Finally, further studies should consider that the Strength and Difficulties Questionnaire, which is often used and validated to measure child behavioral problems, may not be the best instrument to use in very deprived contexts such as slums. Some behavioral problems presented in the questionnaire, such as the hyperactivity problems, were too abstract for low-educated mothers living in slum conditions whose prominent child-related problems might be related more to physical health problems or basic needs related issues like hunger and general health.

## 5. Conclusions

This study contributes to the limited available literature on the role of maternal religiosity in parenting and child outcomes, particularly in a Muslim Arabic context. Maternal religiosity was not directly related to harsh physical parenting. This finding is interesting in a culture where religion is a salient part of people’s life. More research needs to be done to investigate other factors that can play a role in individual differences in harsh physical parenting in the Yemeni slum context. Our results emphasize the role of maternal religiosity as a moderator in the relation between harsh physical parenting and child outcomes, with higher religiosity exacerbating the negative effects of harsh physical parenting on children. The mechanism underlying this moderation needs to be explored in future research. Moreover, for parents with higher levels of religiosity, parenting skills can be addressed as a target for interventions aimed at promoting positive parenting by bodies that work directly with mothers in slum areas like local NGOs, child clinics, and public organizations, etc. This recommendation comes in line with previous research which showed that besides parenting practices, culture plays a significant role in parenting [47]. This can be done by relating positive parenting skills to religious teachings that highlight warmth, support, and care towards children as for instance in this Hadith about the prophet’s sensitivity to children “The Prophet kissed his grandson Al-Hasan bin ‘Ali in the presence of Al-Aqra’ bin Habis. Thereupon he remarked: “I have ten children and I have never kissed any one of them”. Messenger of Allah looked at him and said, “He who does not show mercy to others will not be shown mercy” [76].

## Figures and Tables

**Table 1:** Descriptives of maternal harsh physical interference, religiosity variables, and child behavioral problems (*N* = 62).

Variable	*%*	*M*	SD	Possible Min	Possible Max	Actual Min	Actual Max
(1) Maternal harsh physical interference (present)	23						
(2) Maternal religiosity *		1.05	0.98	0	3	0	3
Religiosity-based vignettes ª		0.72	0.74	0	2	0	2
Religiosity-based parenting goal	32	0.32	0.47	0	1	0	1
(3) Child behavioral problems		2.00	0.27	1	3	2	3

* Sum score of vignettes and parenting-goal questions. ª Higher scores indicate more frequent mention of religion.

**Table 2:** Correlations between maternal harsh physical interference, religiosity variables, and child behavioral problems.

Variable	1	2	3	4	5
Maternal harsh physical interference	—				
Maternal Religiosity sum score	−0.025	—			
Religiosity-based Vignettes ª	−0.079	0.885 ^**^	—		
Religiosity-based parenting goal	0.082	0.690 ^**^	0.274 ^*^	—	
Child behavioral problems sum score	0.127	0.065	−0.026	−0.071	—

** *p* < 0.01 (2-tailed), * *p* < 0.05 (2-tailed), ª Higher scores indicate more religion mentioned.
